# Killing a Killer: What Next for Smallpox?

**DOI:** 10.1371/journal.ppat.1000727

**Published:** 2010-01-29

**Authors:** Grant McFadden

**Affiliations:** Department of Molecular Genetics and Microbiology, College of Medicine, University of Florida, Gainesville, Florida, United States of America; The Fox Chase Cancer Center, United States of America

## Introduction

Now that the 20th century has passed into the domain of history books, we can retrospectively begin to assess the relative contributions that the many advances in the realm of infectious disease have actually made to public health in general. At the top of this virtuous list will surely be the discovery of antibiotics in the 1930s and the use of vaccination to eradicate smallpox as an extant human disease in the 1960s and 1970s. As clearly pointed out in a recent book by D. A. Henderson, one of the leaders of the global smallpox eradication program, this task of ridding *Homo sapiens* from the curse of this ancestral disease was neither easy nor without controversy [1]. In fact, the history of the many consequences of smallpox on humankind reads like a long litany of human misery and calamitous events, but is juxtaposed with the more noble accomplishments that began with the discovery of vaccination by Jenner in 1798 and culminated with the World Health Organization (WHO) certifying the world free of smallpox in 1980 [2]. With this singular accomplishment, as many as 60–100 million individuals who would have been predicted to die of smallpox have been spared from a truly gruesome death. Nevertheless, as is intimated by the timeline in Table 1, which summarizes the history of smallpox and the orthopoxvirus that caused the disease (variola virus), the narrative of smallpox did not stop with its eradication as a pandemic human disease. Instead, we find ourselves still wrestling with an issue that intermingles public health policy, philosophy, national security, and bioterrorism, and affects our perceptions of research ethics with extreme pathogens in general. It boils down to a not-so-simple question: What exactly should the Victor do with the Vanquished?

**Table 1:** History of Smallpox: Timeline of a Serial Killer.

>2000 B.C.	Smallpox appears in humans in Africa and the Far East
1157 B.C.	Pharaoh Ramses V dies of smallpox
910 A.D.	Clinical disease first described (by Rhazes)
1096–1291	Crusaders accelerate smallpox importation to Europe
1507–1530	Aztec, Mayan, and Inca empires decimated by smallpox
1400–1800	European fatalities alone exceed 500 million/century
1763	First intentional use as a bioweapon (against Native Americans)
1798	Vaccination introduced by Jenner
1965	WHO initiates intensified worldwide eradication program
1977	Last natural case of smallpox (in Somalia)
1978	Last case of smallpox in humans (lab accident in the UK)
1980	WHO certifies worldwide eradication of smallpox
1983	All known variola stocks transfered to the two certified WHO collaborating centers (US and Russia)
1993	Variola DNA genome sequence published
1996	World Health Assembly (WHA) recommends variola destruction (in 1999)
1999	WHA recommends postponing destruction to permit further research with live variola virus
1999	First IOM report on research needs for live variola virus
1999	*Biohazard* published (K. Alibeck)
2001	US announces postponement of variola destruction
2009	Second IOM Report on research needs for live variola virus
2011	WHA vote on destruction of the declared live variola virus stocks (expected)

In 1980, this question seemed simpler than now. Following the smallpox eradication, all declared stocks of the live variola virus were rounded up and distilled into two WHO-approved repositories, now residing at the Centers for Disease Control (CDC), Atlanta, United States of America, and at Vector, Novosobirsk, Russia. WHO convened a standing committee to oversee these repositories and issue regulatory approval for any research studies that utilized the live virus stocks at the two sites, with the tacit assumption that the only justifiable long-term fate for these stocks was an autoclave. Then, the revelation that variola virus had been covertly weaponized and stockpiled by the Soviet military [3],[4] led to escalating waves of mistrust and suspicion amongst politicians, government officials, scientists, and health policy experts alike [5]. Factions then formed, with the two sides collectively promulgating an agenda that was either pro-destruction or anti-destruction, and cogent arguments were made by members of both camps as to why the declared stocks of variola virus should be maintained or not [6]–[8]. In the meantime, the member states holding the declared stocks of live virus (i.e., the US and Russia) held their own internal deliberations of what to do next, in a kind of *pas de trois* with the WHO that continues to this day. In the case of the US, input was sought from the Institute of Medicine (IOM), which has struck two expert committees (the first issued its report in 1999, and the second committee report was released in July 2009 at http://www.iom.edu/Reports/2009/LiveVariolaVirusContinuingResearch.aspx; [9]) on the scientific rationale for any further research that would require live variola virus. It is expected that these two IOM reports will be factored into the US decision as to how to respond to any future request from WHO (expected in 2011), following a vote of member states of the World Health Assembly on the specific issue of whether the declared live variola virus stocks held at both sites should now finally be destroyed.

## But Science Continues to March On

In the meantime, particularly in the past decade, some genuinely intriguing science has been conducted with variola virus and closely related pathogenic orthopoxviruses. Many diverse scientific fields that impinge directly on the issue of variola virus research potential (e.g., genomics, proteomics, virus–host interactomics, bioinformatics, synthetic biology, etc.) have been moving forward at breakneck speed, and so has the technical ability to query related issues like viral pathogenesis and host tropism. The nearly complete genomic sequences of two variola isolates were first published in the early 1990s, but now that almost 50 distinct genome sequences are available on the Web (http://www.poxvirus.org), derived from independent isolates collected throughout the world at various times in the 20th century, new clues as to the origin, spread, and evolution of variola clades within the human population have been deduced [10],[11]. We now know that variola virus is most closely related genetically to two tightly host-restricted orthopoxviruses, the camel-specific camelpox and the gerbil pathogen taterapox, neither of which infect humans. In contrast, the one orthopoxvirus that can cause a clinical disease in humans that most closely resembles smallpox is spread to humans by zoonotic infection with an African rodent virus called monkeypox virus. However, monkeypox is genetically much more diverged from variola virus and likely represents a distinct lineage of orthopoxviruses.

Although variola virus does not infect nonhuman primates, some aspects of late-stage smallpox disease can be modeled in macaques, provided the virus is administered intravenously at high doses [12]. In microarray studies with such variola virus-infected macaques, it has been shown that this virus has learned how to turn off the host systemic inflammatory responses that are under the control of tumor necrosis factor (TNF) and nuclear factor kappa B (NFκB) in vivo [13]. However, it is important to appreciate the important caveat that variola virus in nature is restricted to only human hosts, and no surrogate nonhuman primate accurately models smallpox disease, either in terms of infectious doses required to initiate infection or in disease progression. In fact, this limitation means that animal models may never be able to completely mimic smallpox disease in humans.

In the past decade, various proteomic strategies in vitro have revealed that the variola genome encodes many potent inhibitors of various human immune response cascades, including targets such as serum complement, IL-18, interferon-gamma, TNF, chemokines, and various signaling cascades [14]–[16]. Most recently, systematic yeast 2-hybrid screening of the unique variola proteins against the entire human proteome has uncovered even more viral modulators of human immune signaling, including a new poxviral inhibitor family that targets a precursor NFκB1 protein [17]. In fact, there is every reason to suspect that many more secrets about human “anti-immunology” remain undeciphered and undiscovered within the variola genome. What remains contentious is whether live variola virus will ever be required in order to unravel these secrets. We simply cannot predict whether future development of more “humanized” small animal models might progress to the point where smallpox could be more accurately modeled outside of human hosts.

In addition, biodefense-driven research efforts that were sparked by fears of the potential re-emergence of smallpox have also generated new classes of potent anti-poxviral drugs, such as ST-246 and the lipid-soluble cidofovir derivative CMX001, and newer generations of vaccines that are more compliant with regulations of the Food and Drug Administration (FDA) (e.g., ACAMBIS 2000) or safer for immunocompromised individuals (e.g., MVA or LC16m8) have been developed and stockpiled. These drugs and vaccines have the dual benefit that they are likely also efficacious for other related zoonotic orthopoxvirus infections of humans, particularly monkeypox and cowpox viruses. Additionally, the new anti-poxviral drugs have been used to treat rare cases of runaway infections with the live vaccinia vaccine itself. Newer diagnostics based on PCR techniques or directed sequencing have refined the ability to distinguish bona fide variola infections from those caused by closely related orthopoxviruses. In fact, these advances can be counted among the genuine success stories made possible by the increased biodefense funding in the US since the terrorism events of 2001.

But despite these advances, there is far more that we simply do not understand about smallpox disease or its causative virus. The smallpox vaccine, vaccinia virus, remains the poster-child for human vaccines, but we have only begun to understand how vaccinia-induced immune responses protect vaccinees from orthopoxvirus infections [18],[19]. We do know that both memory B cell and T cell immune responses combine to provide the disease protection conferred by the live vaccine. Specific combinations of vaccinia proteins within subunit vaccines have also been shown to be capable of inducing protective immunity via specific antibodies or T cell responses in animal models of orthopoxvirus disease. In contrast, we still do not understand why smallpox disease was so lethal in humans, or if host responses such as the oft-quoted and still poorly-understood “cytokine storm” is really a key instrument of the disease pathophysiology. In fact, we do not comprehend the basis for the strict host tropism of variola virus for humans, nor why there are no animal reservoirs. So, there is really no scientific debate about whether variola virus still has much to teach us about human immunology and viral pathogenesis in general. Instead, the main flashpoint for debate remains the issue of risk versus benefit at acquiring any more scientific information with live variola virus. More recently, however, another confounding element has entered this debate that may soon render the issue of retention versus destruction moot. Specifically, can we actually ever truly get rid of this virus?

## Vanquished Perhaps, but Defeated?

In the 1980s, the debate focused on whether the destruction of the declared variola stocks would actually free the planet forever from the specter of smallpox re-emergence, or whether destruction would simply make the world a more dangerous place where suspected covert stocks of virus would assume greater danger as potential bioweapons or agents of bioterrorism. Even now in the first decade of the 21st century, we still do not know if any live variola virus stocks exist outside of the two WHO-approved repositories, but the combination of an extensive public literature on variola virus genomic sequences coupled with the rapidly advancing technologies of DNA gene synthesis and synthetic biology have now made the possibility of creating live variola virus (and indeed any viral pathogen) from scratch readily achievable.

Although variola virus remains the first and only human pathogen to sit in the gallows, waiting potential execution, it will not be the last. Polio stands a reasonable chance of being eradicated as a human disease in our lifetime (and Rinderpest as a cattle disease), but as Wimmer's lab showed in 2002, any scientist with access to a gene synthesizer can now construct live polio virus using relatively standard laboratory reagents [20]. Nobody has yet published the complete construction of a live poxvirus from fully synthetic genes, but the technologies needed for resuscitating live poxviruses from plasmids, PCR amplicons, or bacmid fragments are now well-established [21]–[23]. In fact, the only two remaining ingredients now needed to create a live poxvirus from elemental chemicals are motivation and money. This bedeviling issue of how synthetic biology can be applied to human pathogens has not escaped attention by scientists and policymakers alike, and the debate becomes only more problematic as the technologies for synthetic biology increase in robustness and decrease in cost [24],[25].

## What to Do?

Given the above conundrum, the obvious question is whether the destruction of the declared live variola virus stocks would be a genuine victory for humankind or merely be a symbolic gesture that provides only an illusion of security. Should the message to the scientific community of the future be that no further scientific queries will be tolerated that require live variola virus? Presently, the WHO-mandated restrictions on labs working even with noninfectious plasmid DNAs containing variola gene sequences are very stringent [26], but advancing genomic and proteomic technologies remain far ahead of legal restrictions. For example, it is still undefined what constitutes a “legal” variola gene sequence. Some orthopoxviruses, like vaccinia virus, encode many genes that are essentially identical to variola virus, or that can be easily mutated into genes that express the orthologous variola protein exactly. Furthermore, synthetic genes, particularly those that are codon-optimized, can be created that are very different in terms of nucleotide sequence from the native variola genome sequence, but can be translated into accurate variola proteins. Indeed, if a synthetic poxvirus were created that possessed only codon-optimized genes for maximal efficiency in human cells, we simply do not know if it would be pathogenic in vivo or whether it would be transmutated into a less virulent version of its parent. Presently, any experiment involving the genetic manipulation of variola virus, including even the cassetting of individual variola genes into another poxvirus, is strictly forbidden (WHO policies can be found at http://www.who.int/csr/disease/smallpox/research/en/index.html).

Similar ethical issues remain when we consider the likelihood of whether a new recombinant orthopoxvirus, derived from poxviruses that alone are nonpathogenic to humans, might be capable of causing smallpox-like disease in humans. It is already known that zoonotic infections with monkeypox virus resemble clinical smallpox closely, but these infections are only poorly transmissible from human to human [27],[28]. It is still impossible to predict the biologic or pathogenic properties of novel poxviruses created when closely related poxviruses recombine with each other, which can and does occur either in the wild or in a laboratory.

**Figure 1:**
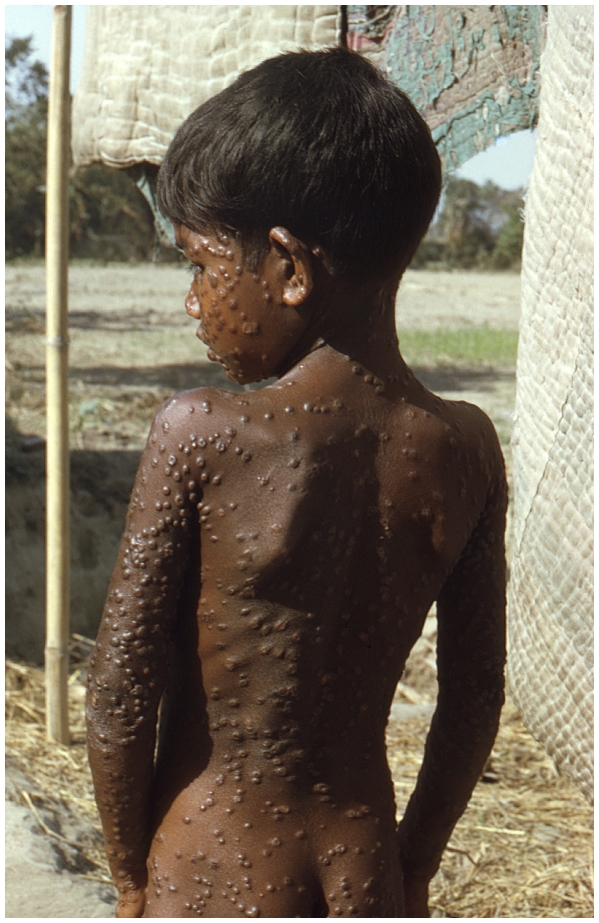
Smallpox is a uniquely human disease. This 1974 photo of a young villager in the Rangpur district of northeastern Bangladesh depicts one of the last known infections of a human with variola major virus. (Source: Jean Roy, Emory Global Health Institute, from the CDC Public Health Image Library at http://phil.cdc.gov/phil/home.asp.)

Ultimately, the reason this debate remains contentious is that variola virus has killed more human beings in the civilized era than any other known pathogen. Even though the disease itself has not been seen for over 30 years, pictures of its victims still have the power to remind us of why this viral pathogen is still feared (Figure 1). No civilized person wants to see another smallpox case again in humans [29], but exactly what is the surest route to that end?

The debate about the potential destruction of variola virus, for better or worse, is returning to the front page. Now, however, the emergence of open access publishing and open source technology allows for more input and dialogue from a wider spectrum of people who may wish their views to be registered. The member states of the World Health Assembly will soon be polled for their vote on whether the existing declared stocks of variola virus should be destroyed or not. So, until then, the readers of *PLoS Pathogens* are invited to comment on this issue online via the Comments tab, which is located underneath the article title (commenting requires a PLoS Journals account; read more at http://www.plospathogens.org/static/help.action#account).

The debate may also prove to be instructive when the next human microbial pathogen lands on death row, awaiting our collective verdict.
